# The quality of the sickness certificate. A case control study of patients with symptom and disease specific diagnoses in primary health care in Sweden

**DOI:** 10.1080/02813432.2019.1639905

**Published:** 2019-08-13

**Authors:** Karin Starzmann, Per Hjerpe, Kristina Bengtsson Boström

**Affiliations:** a Department of Public Health and Community Medicine, Primary Health Care, the Sahlgrenska Academy, University of Gothenburg, Gothenburg, Sweden;; b R&D Centre Skaraborg Primary Care, Skövde, Sweden

**Keywords:** Sick leave certification, general practice, diagnoses, medically unexplained symptoms, guideline adherence

## Abstract

**Objective:** To compare information in sickness certificates and rehabilitation activities for patients with symptom diagnoses vs patients with disease specific diagnoses.

**Design:** Retrospective case control study 2013–2014.

**Setting:** Primary health care, Sweden.

**Subjects**. Patients with new onset sickness certificates with symptom diagnoses *n* = 222, and disease specific diagnoses (controls), *n* = 222.

**Main outcome measures:** Main parameters assessed were: information about body function and activity limitation in certificates, duration of sick leave, certificate renewals by telephone, diagnostic investigations, health care utilisation, contacts between patients, rehabilitation coordinators, social insurance officers, employers and occurrence of rehabilitation plans.

**Results:** Information about body function and activity limitation was sufficient according to guidelines in half of all certificates, less in patients with symptom diagnoses compared to controls (44% vs. 56%, *p* = 0.008). Patients with symptom diagnoses had shorter sick leave than controls (116 vs. 151 days *p* = 0.018) and more certificates issued by telephone (23% vs. 15% *p* = 0.038). Furthermore, they underwent more diagnostic investigations (32% vs. 18%, *p* < 0.001) and the year preceding sick leave they had more visits to health care (82% vs. 68%, *p* < 0.001), but less follow-up (16% vs. 26%, *p* < 0.008). In both groups contacts related to rehabilitation and with employers were scarce.

**Conclusion:** Certificates with symptom diagnoses compared to disease specific diagnoses could be used as markers for insufficient certificate quality and for patients with higher health care utilisation. Overall, the information in half of the certificates was insufficient and early contacts with employers and rehabilitation activities were in practice missing.

**KEY POINTS**
- Symptom diagnoses are proposed as markers of sickness certification quality. We investigated this by comparing certificates with and without symptom diagnoses.
- Certificates with symptom diagnoses lacked information to a higher degree compared to certificates with disease specific diagnoses.
- Regardless of diagnoses, early contacts between patients, rehabilitation coordinators and social insurance officers were rare and contacts with employers were absent.

## Introduction

Paid sick leave is a hallmark of most welfare states [1]. Sickness benefits in Sweden are tax-funded and related to the income of the individual. Because of nationally rising costs of sickness benefit during 1997–2004 [2], the Swedish government made efforts to lower the cost and improve the quality of the sick leave process. Among other things, extensive education was provided to physicians, starting in 2006 and a decision support with guidelines for sickness certification was issued by the National Board of Health and Welfare in 2007 [3]. A new role, the rehabilitation coordinator, mainly occupational therapists, physiotherapists, or nurses was introduced in health care, to facilitate the rehabilitation and the communication between the patient, the employer and the social insurance officer [4]. The cost of sickness benefit has fluctuated during recent decades. After a decline from 2003 to 2010, there was a rise until 2017 after that the costs have stabilised.

The sickness certificate is the main means of communication between physicians and social insurance officers. The completeness and quality of the information given in the certificate is therefore crucial for a correct assessment of the patient’s ability to work and eligibility for economic support [5]. The certificate is intended to be filled out according to a template, where the *D*iagnosis, the impairment of body *F*unctions and the *A*ctivity limitations are logically linked in the so-called “DFA chain”. It has been shown that a majority of certificates have missing information, but also that the information does not confirm the physicians’ objective evaluation of the patient [6]. The diagnoses on the certificate are of specific importance, as the recommendations from the National Board of Health and Welfare on eligibility for and duration of sick leave are based on and differ between diagnoses [3]. Adequate information in the sickness certificate is also critical to ensure that the patient receives appropriate coordinated rehabilitation.

Symptom diagnoses, R0.00–R99.9, are defined according to ICD-10, chapter 18 [7]. They are used during the diagnostic process before a specific diagnosis is established. They can also include medically unexplained physical symptoms (MUPS) and medically unexplained symptoms (MUS), when extensive investigations fail to result in a specific diagnosis [8,9]. According to the recommendations, available from the National Board of Health and Welfare that were in effect until 2017, a symptom diagnosis was acceptable for the first 2 weeks of sick leave, after which a disease-specific diagnosis ought to be used. Symptom diagnoses was found to be associated with lower quality of information on the sickness certificate and rehabilitation process over time [10] and a large proportion of certificates lacked important information about function impairment and activity limitations.

Continuous evaluation of the sickness certification over time is of utmost interest to national authorities, and it is important to develop potential quality markers. Therefore, the aim of this study was to evaluate the usefulness of diagnoses on certificates as quality markers by comparing the sickness certificates for patients with symptom diagnoses and patients with disease-specific diagnoses concerning coherence of text in the certificate, rehabilitation activities and health care utilisation.

## Material and methods

The study was conducted in Skaraborg, a rural area in the Västra Götaland Region with approximately 258,500 inhabitants in 2013. The 20 publicly run primary health care centres (PHCC) in the area cared for approximately 75% of the inhabitants.

All sickness certificates recorded in these 20 PHCCs during 2013–2014 were retrieved from the electronic medical records (ProfDoc, Journal III). Patients with sickness certificates recorded in 2012 were excluded in order to include only new onset sick leave cases. All patients with symptom diagnoses (R00.0–R99.9) on the sickness certificate at day 28 were included. For each patient with a symptom diagnosis, one control with a disease specific diagnosis and new onset sick leave of a minimum of 28 days was selected. The controls were matched by sex and date of birth by picking the first consecutive patient without symptom diagnoses after a patient with symptom diagnoses from a list sorted by sex and date of birth.

The patients were included in the study on the day they first visited the physician for the studied sick leave period of 28 days or longer. The sick leave period was defined as the date on the first certificate to the last date on the last recorded certificate. The patients left the study if they were referred to other clinics or if the sick leave was prolonged beyond the introduction of a new electronic medical record system in 2015, precluding further retrieval of data.

Data from medical records and sickness certificates were gathered manually using a predefined protocol. Information was compiled on diagnoses and descriptions of function impairment and activity limitation were coded according to the Swedish version of the International Classification of Function – Child and Young version (ICF-CY) [11] at the second level (e.g. pain = b280, walking = d450). The descriptions were based on the physicians’ assessment of the patient, i.e., observations of the patient’s function impairment or activity limitation (objective), or the patients’ descriptions (subjective). Descriptions of diagnosis, function impairment and activity limitation (DFA chain) were classified as (a) coherent and complete, (b) description of function impairments, (c) description of activity limitations.

Investigations and referrals, number of sickness certificates renewed by telephone, patients’ employment status and occupation, contacts with social insurance officers and rehabilitation activities were recorded. The frequency of referrals and time to contact with other stakeholders (social insurance officers, rehabilitation coordinators and Swedish employment service) were calculated. The number of contacts at the PHCC (physicians, registered nurses and physiotherapists) in the year preceding the sickness certification and also visits to other health care providers were recorded as well as clinical investigations, alcohol audit, and prescriptions of analgesic drugs, opioids, proton pump inhibitors, antidepressants and benzodiazepines.

Descriptive statistics were performed for means and standard deviations. Differences in discrete variables were calculated with Chi^2^ tests, for continuous variables with T-tests and for small samples with the Wilcoxon test. Differences in sick leave duration were calculated with survival analysis with SAS LIFETEST procedure [12]. All analyses were performed in SAS (9.3, Inc., Cary, NC, USA). The level of significance was defined as <0.05.

## Results

**Figure 1.**
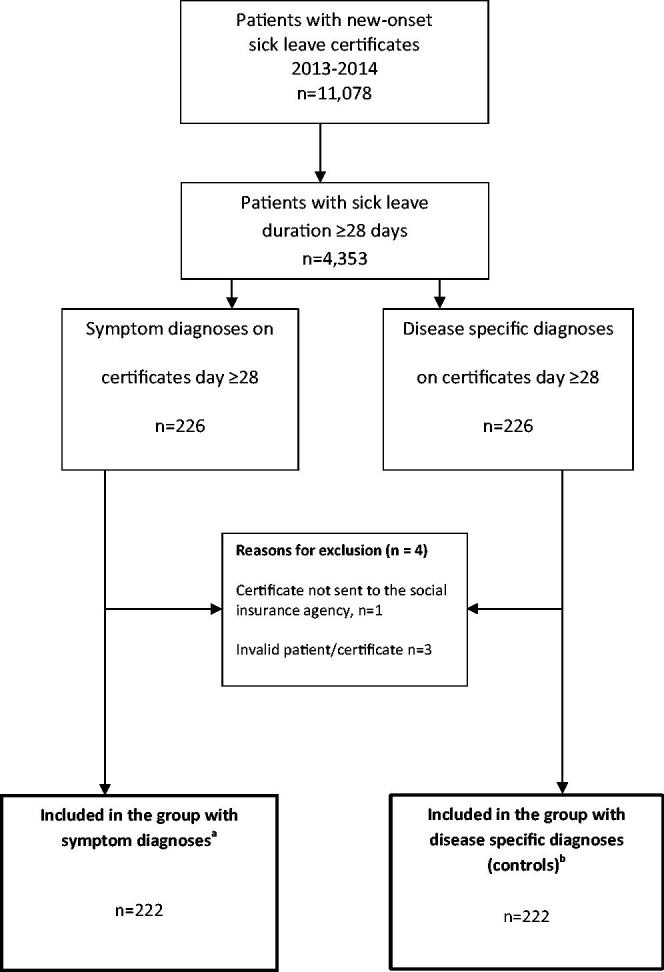
Flow chart of study subjects, patients with symptom diagnoses and controls with disease-specific diagnoses on the sickness certificates. ^a^SD: Symptom diagnosis, ICD-10, chapter XVIII, (R). ^b^Control group: ICD-10, all other chapters.

Two hundred and twenty-two patients in each group were included in the study, Figure 1. There were more women (*n* = 162) than men (*n* = 60), and the women were younger than the men (41 ± 13 vs. 44 ± 13 years, *p* = 0.04), Table 1. Forty-three patients in the group with symptom diagnoses and 50 controls did not have complete data due to referrals to other clinics during follow-up or a sick leave that extended beyond introduction of a new medical record system.

**Table 1.** Characteristics of patients with symptom diagnoses and controls with disease-specific diagnoses.

	Symptom diagnoses *n* = 222, f/m 162/60	Controls *n* = 222 f/m 162/60	*p*
Age women (years)	41 ± 13	41 ± 13	
Age men (years)	44 ± 13	44 ± 13	
Length of sick leave All individuals (days)	116 ± 138	151 ± 171	0.018^a^
Length of sick leave, women (days)	107 ± 125^b^	154 ± 172	0.012^a^
Length of sick leave, men (days)	134 ± 91^b^	150 ± 105	ns^a^
Contact with coordinator before sick leave, *n* (%)	7 (3)	8 (4)	–
Contact with coordinator during sick leave, *n* (%)	10 (4)	19 (9)	ns
Contact with physiotherapist, *n* (%)	59 (27)	63 (28)	ns
Planned rehabilitation, *n* (%)	5 (2)	12 (5)	ns
Social insurance officer contacted the PHCC, *n* (%)	22 (10)	17 (8)	ns
Sick leave rejected, *n* (%)	0	1 (0.5)	–
Certified by telephone, *n* (%)	50 (23)	33 (15)	0.038
X-ray or ultrasound examination, *n* (%)	71 (32)	40 (18)	<0.001
Visit to physician at PHCC preceding year, *n* (%)	183 (82)	151 (68)	<0.001
Visit to emergency clinics and other healthcare providers, *n* (%)	49 (22)	28 (13)	<0.009
Anti-depressants drugs, *n* (%)	49 (22)	97 (44)	<0.001
AUDIT or tests for alcohol, *n* (%)	17 (8)	16 (7)	ns
Planned follow-up by physician, *n* (%)	35 (16)	58 (26)	0.008

Frequencies and duration of different events in the rehabilitation process.

Data are means ± standard deviations or numbers and frequencies.

f: female; m: male; ns: non-significant; SIO: social security officer.

^a^Differences calculated with Kaplan-Meier estimator.

^b^No difference in length of sick leave between women and men with symptom diagnoses.

### Sickness certificates and sick leave

In the group with symptom diagnoses, the most common diagnosis on the sickness certificate on the 28th day of sick leave was “Pain, not otherwise classified” (23%), followed by “Dizziness and giddiness” (14%), “Malaise and fatigue” (14%), and “Abdominal and pelvic pain” (14%). Among the disease-specific diagnoses, psychiatric diagnoses were most common (52%), followed by musculoskeletal diagnoses (26%).

Coherence of the DFA chain between the diagnosis and the description of function impairment and activity limitations was found in only half of the certificates, to a lesser degree in the group with symptom diagnoses compared to the controls (44% vs. 56% *p* = 0.008), Table 2. The physicians described fewer objective findings in medical status and impairment of functioning in the group with symptom diagnoses (24% vs. 45% *p* < 0.001).

**Table 2.** Analysis of the text under the predefined subheadings in the sickness certificates spanning the 28th day of sick leave.

	Symptom diagnoses *n* = 222, f/m 162/60	Controls *n* = 222, f/m 162/60	*p*
Coherent DFA chain, all^a^	97 (44%)	125 (56%)	0.008
Coherent DFA chain, women^a^	65 (40%)	88 (54%)	0.02
Coherent DFA chain, men^a^	32 (53%)	37 (62%)	ns
Description of activity limitations, all	135 (61%)	140 (63%)	ns
Activity limitations are described women	94 (58%)	99 (61%)	ns
Activity limitations are described men	41 (68%)	41 (68%)	ns
Impairment of body functions described all	215 (97%)	220 (99%)	ns
Objective evaluation of impairment of functioning, all	53 (24%)	100 (45%)	<0.001
Objective evaluation of impairment of functioning, women	40 (25%)	66 (41%)	0.003
Objective evaluation of impairment of functioning, men	13 (22%)	34 (57%)	<0.001
Presence of explanation for sick leave duration exceeding decision support	8 (4%)	4 (2%)	
Number of described impairments of body functions^b^	4.4 ± 2.6 (0–14)	4.5 ± 2.5 (0–12)	0.86
Number of described activity limitations^b^	1.7 ± 1.7 (0–9)	1.9 ± 1.9 (0–10)	0.28

DFA: Diagnosis, impairment of body Functions, Activity limitations; f: female; m: male; ns: non-significant.

^a^Coherent DFA chain, presence of association between descriptions of D, F and A in the certificate according to a predefined evaluation form.

^b^Classified in the ICF-CY at the second level (e.g. pain = b280, walking = d450) in four-piece code. Each code can only occur once for each patient.

The duration of sick leave for patients with symptom diagnoses was shorter than in the controls (116 ± 138 vs. 151 ± 171 days, *p* = 0.018) but sickness certification by telephone was more common in this group (23% vs. 15%, *p* = 0.038). In certificates renewed by telephone coherence in the DFA-chain did not differ between the groups or between men and women.

### Health care consumption

Patients with a symptom diagnosis visited physicians at the PHCC more often during the year before the sickness certification compared to the controls (82% vs. 68%, *p* < 0.001). They also visited emergency clinics and other health care providers to a greater extent (22% vs. 13%, *p* = 0.009) and had more X-ray or ultrasound examinations (32% vs. 18%, *p* < 0.001). However, there was neither a difference in the use of alcohol audit, prescription of analgesic drugs, opioids, proton pump inhibitors or benzodiazepines between the groups, nor in the number of visits to registered nurses and physiotherapists. On the other hand, fewer were treated with anti-depressants in the group with symptom diagnoses compared with the controls (22% vs. 44% *p* < 0.001) and they had fewer visits due to mental complaints (6% vs. 14%, *p* = 0.005).

### Rehabilitation activities

Planned rehabilitation was rarely mentioned in the certificates with no difference between the groups, but follow-up was less common in the group with symptom diagnoses compared to the controls (16% vs. 26%, *p* = 0.008). Further, it was rare that the staff at the PHCC was contacted by the social insurance officers, regardless of group (10% vs. 8%, ns) and there was a great variation in duration to the first contact (range 43–381 vs. 35–790 days, ns). In addition, rehabilitation meetings were rare in both groups (7% vs. 10%, ns), and meetings with the Swedish employment service were even less common (2% vs. 1%). The rehabilitation coordinator contacted very few patients (4% vs. 9%, ns), with a wide variation in time (13–770 days vs. 9–460 days). The employer was only contacted in one case.

## Discussion

The main finding in this study was that the information necessary for assessment of sickness benefit eligibility and need for rehabilitation (DFA chain) was found in only half of the certificates and significantly less so for patients with symptom diagnoses compared to controls. In patients with symptom diagnoses the duration of sick leave was shorter and more certificates were renewed by telephone. Further, they visited PHCC, hospitals and emergency clinics more often before the sick leave period and had more diagnostic procedures compared to controls. Contact with other stakeholder were rare and usually late in both groups and contact with the patient’s employer was only found in one instance.

### Strengths and weakness of the study

One strength of this study was that the data was retrieved from medical records directly and without selection, mirroring the current clinical practice. Further, the design with matched controls to patients with symptom diagnoses made comparison between the groups possible. Only new onset sick leave cases were studied in order to avoid patients with previous long term sick leave to be included. The patients were followed the year before the sickness certification in order to compare the groups with respect of health care consumption. A weakness was that the follow-up ended in 2015 when a new computerised medical journal system was introduced so that patients with longer sick leave could not be followed. Further, only one ICD-10 code, the main code on the certificate, was studied, other diagnoses occurring simultaneously could of course play a role in the physician´s assessment. We studied factors presumed by us to be of importance for sick leave certification and that could differ between the groups, other factors not included in this study could also play a role.

### Findings in relation of other studies

We found a difference in completeness of sickness certificates between patients with symptom diagnoses and controls in line with the results earlier described [10]. This makes certificates issued after more than 4 weeks with symptom diagnoses useful as a marker for incompleteness of information and for patients that might be in need of extra attention because of their increased health care utilisation. Equally important though, was that in both groups only half of the certificates had sufficient information to make assessment of sickness benefit eligibility and planning of rehabilitation possible. A trend of increasing information on certificates over time was shown from 2004 to 2009 when it reached 50% [10], the level we describe in the current study. The authors suggest reminders, compulsory certificate fields and structured guidance for improving the quality of sickness certificates. In another study from Northern Sweden in 2015 an even lower frequency of complete sickness certificates was described [5] and more information on and education in the use of ICF was proposed. Since these studies were performed electronic certificates with compulsory field and direct access to the decision support have been introduced and education in the use of ICF has been performed for the majority of physicians in primary care in Skaraborg. This did not seem to have had much impact on the information on certificates as the level of sufficient information did not increase. A recently published study of interviews of GPs in Sweden revealed that physicians use unsanctioned techniques for having sickness certificates accepted [13]. These findings highlights the need of other means to improve the information on the certificate. From 2019 an extended electronic support will be introduced offering the physician a set of ICF terms associated to the selected diagnosis on the sickness certificate. However, the physicians will still have to evaluate the patients’ activity limitation in relation the to the patient’s specific work conditions. This is something physicians are not comfortable doing as they often lack detailed knowledge and understanding of the working conditions [14]. As evaluation of body function impairment corresponds to medical terminology it is more straightforward for the physicians compared to activity evaluation. These assessments are described as problematic also in other counties such as Holland [15]. In Germany, the Mini-ICF-APP, an observer rating for the description of activity and capacity status is widely used for description of work ability (impairment) [16,17] and is suggested for use in social-medicine practice by the German Statutory Pension Insurance.

In the current study, regardless of diagnoses on the certificate, there were almost no contacts with the patient’s employer and the rehabilitation plans were scarce. Workplace interventions have been described to reduce the duration of sick leave and facilitate return to work [18,19]. Work anxiety, a condition that might reduce the patient's inclination to return to work has recently been acknowledged [17]. Increased cooperation with the employers and paying attention to work anxiety and thus supporting the patient by social interaction training and work adjustment [17,18] might reduce the duration of sick leave. This could be a next step to facilitate rehabilitation as described in a study protocol for patients with stress-related conditions [20].

The diagnosis on the certificate is decided by the physician during the encounter with the patient and in some cases the symptoms are not yet explained by a disease. Assigning a symptom diagnosis can be helpful to avoid over diagnosis which could be inappropriate both in terms of treatment and for the patient´s perception of their illness. Some patients in the group with symptom diagnoses probably had MUPS or MUS as an increased health care consumption could be seen in this group as well as more referrals and investigations but not active follow-up to the same extent as in the controls. This indicates that at least some patients in the group with symptom diagnoses have complicated health problems and a disease specific diagnosis, which is easier to accept, might not be possible to determine. This is a challenge to the relation between patient and doctor [21–23]. Therefore, it might be valuable to recognise these patients early as they are at risk for longstanding sick leave [24,25]. In the Netherlands specifically trained specialists take care of patients with MUS and MUPS according to management guidelines [26].

Some findings in our study surprised us. First, it was rare that the social insurance officers sent questions about the sickness certificate to the physician. This may have changed with the more restrictive assessment policy introduced after this study was performed. Secondly, patients with symptom diagnoses had shorter sick leave than controls. One explanation might be that the social insurance agency in Sweden has nowadays become stricter in their assessment of eligibility for sick leave and this could negatively affect groups with less well-defined illnesses. It is therefore important to increase the awareness that the certificates with symptom diagnoses need to be more carefully written, especially the description of physical and mental limitations in relation to the patients working situation. Another explanation might the large proportion of depression among the controls as this condition usually generates long sick leave. This was supported by the finding of increased prescription of anti-depressant drugs in the controls compared to the group with symptom diagnoses. It is well-known that psychiatric illnesses are most expensive for the society [27] and it causes the highest frequency for sick leave according to our earlier study [28]. The shorter duration of sick leave connected to symptom diagnoses could also be explained by less serious conditions in some patients leading to a quicker recovery.

The social insurance agency recommends that sickness certificates should not be renewed by telephone. We found that certificates were issued by telephone to a greater extent in the group with symptom diagnoses compared to those in the controls. Despite this, we found no association between completeness of information on the DFA chain in either group and telephone renewals compared to renewals during visits to the PHCC. As more and more healthcare is provided by telephone and over the internet we suggest revisiting recommendations regarding the mode of renewals.

## Meaning of the study

This study showed that certificates with symptom diagnoses lacked sufficient information to a greater extent than in certificates with disease specific diagnoses and patients with symptom diagnoses had a higher health care utilisation. This could help identify patients in need of more attention in the sick leave process and evaluation of function and activity limitations as well as medical needs. Of note, around half of the certificates in both groups lacked sufficient information and contacts with other stakeholders in the rehabilitation were sparse. An increase in early contacts for rehabilitation and with employers would be desirable. Using telephone renewals of sick leave did not affect the information in the sickness certificate compared to visits to the PHCC.
